# Portuguese validation of the Internet Addiction Test: An empirical study

**DOI:** 10.1556/JBA.3.2014.2.4

**Published:** 2014-06-13

**Authors:** HALLEY M. PONTES, IVONE M. PATRÃO, MARK D. GRIFFITHS

**Affiliations:** ^1^International Gaming Research Unit, Nottingham Trent University, Nottingham, United Kingdom; ^2^Unidade de Investigaääo em Psicologia e Saúde, ISPA – Instituto Universitário, Lisbon, Portugal

**Keywords:** Internet addiction, online addiction, Internet Addiction Test, Portuguese samples, student populations

## Abstract

*Background and aims:* Research into Internet addiction (IA) has increased greatly over the last decade. Despite its various definitions and general lack of consensus regarding its conceptualisation amongst researchers, instruments for measuring this phenomenon have proliferated in a number of countries. There has been little research on IA in Portugal and this may be partly due to the absence of standardised measurement tools for assessing IA. *Methods:* This study attempted to address this issue by adapting a Portuguese version of the Internet Addiction Test (IAT) via a translation-back translation process and Confirmatory Factor Analysis in a sample of 593 Portuguese students that completed a Portuguese version of the IAT along with questions related to socio-demographic variables. *Results:* The findings suggested that the IAT appears to be a valid and reliable instrument for measuring IA among Portuguese young adults as demonstrated by its satisfactory psychometric properties. However, the present findings also suggest the need to reword and update some of the IAT’s items. Prevalence of IA found in the sample was 1.2% and is discussed alongside findings relating to socio-demographic correlates. Limitations and implications of the present study are also discussed. *Conclusions:* The present study calls for a reflection of the IAT while also contributing to a better understanding of the basic aspects of IA in the Portuguese community since many health practitioners are starting to realise that Internet use may pose a risk for some individuals.

## INTRODUCTION

Research studies dating back to the mid-1990s first suggested the potential for harmful consequences resulting from the excessive use of the Internet (Brenner, 1997; Greenfield, 1999; Griffiths, 1999; O’Reilly, 1996; Young, 1998a). These studies suggested that social pathologies were beginning to surface the cyberspace (Griffiths, 1996). The emergence of so-called ‘Internet Addiction’ (IA) in seminal case studies (Griffiths, 2000a; Young, 1996) clearly showed that there was the need for a valid measurement tool of this new phenomenon.

Nevertheless, research on IA has witnessed a significant proliferation over the past decade (Kuss, Griffiths, Karila & Billieux, 2014) that contributed greatly to the scientific debate prior to the publication of the latest fifth edition of the *Diagnostic and statistical manual of mental disorders* (DSM–5; American Psychiatric Association [in the following APA], 2013) as to whether or not IA should have been included as a separate disorder (Block, 2008; Petry & O’Brien, 2013; Pies, 2009). Similarly, Griffiths (2000b) raised important questions concerning the nature of the addiction itself asserting that it was important to differentiate between addictions *on* the Internet and addictions *to* the Internet. One of the main implications of this assertion was that researchers should not only investigate generalised Internet use but also be aware that many excessive users are not ‘Internet Addicts’ but just use the Internet excessively as a medium to fuel other addictions (Griffiths, 1999). For instance, he argued that online gambling and gaming addicts are not Internet addicts but simply use the Internet as the most convenient medium to engage in their primary addiction. Therefore, they are not addicted to the Internet *per se*.

Despite the debate and controversies, the DSM Substance Use Disorder Working Group decided – for the first time – to include a behavioural addiction (i.e., Internet gaming disorder) in Section 3 of the DSM-5 (APA, 2013) as a mental condition that needs further studies before inclusion into the main text. Recognition by official psychiatric bodies of behavioural addiction represents a major change in the conceptualisation of addiction and is likely to lead to an increase in research into addictions both on and to the Internet (e.g., Internet gaming disorder) worldwide (Griffiths, King & Demetrovics, 2014).

In regard to the instruments for assessing IA, the Internet Addiction Test (IAT) was developed by Kimberly Young as one of the first proposed assessment tools for measuring IA (Young, 1998b). Despite being developed in a book as a form of a quiz without any psychometric validation, it has been extensively used and adopted by researchers all over the world (e.g., Christakis, Moreno, Jelenchick, Myaing & Zhou, 2011; Dong, Lu, Zhou & Zhao, 2011; Ghassemzadeh, Shahraray & Moradi, 2008; Liberatore, Rosario, Colón-De Marti & Martínez, 2011; Morrison & Gore, 2010; Razieh, Ali, Zaman & Narjesskhatoon, 2012), even though its first psychometric validation only occurred in 2004 (Widyanto & McMurran, 2004) making this test the first one to be validated for measuring IA (Young, 2011). Since this first validation study, the IAT has been systematically adapted in various countries such as Italy (Ferraro, Caci, D’Amico & Di Blasi, 2007), France (Khazaal et al., 2008), United States (Jelenchick, Becker & Moreno, 2012), Cyprus (Panayides & Walker, 2012) and more recently in Lebanon (Hawi, 2013).

It should also be noted that the IAT had its roots from an earlier study by Young (1998a) where she conducted a brief exploratory study that attempted to investigate the possible existence of IA and the extent of the problems caused by Internet misuse. Here, IA was assessed by a questionnaire comprising eight questions adapted from the DSM-IV criteria for pathological gambling (APA, 1994). Participants that endorsed five (or more) of the eight criteria were classed as Internet addicted users. Other authors suggested different cut-off criteria for Young’s scale (e.g., Beard & Wolf, 2001; Dowling & Quirk, 2009). At a later stage, Young devised the IAT with the addition of 12 items (Young, 1998b).

To the authors’ knowledge, no study of IA has ever been carried out in Portugal. Validating the IAT for a Portuguese student sample arguably represents a potentially major step in advancing the field of IA in Portugal. Therefore, the aim of the present study was twofold. Firstly, to develop a Portuguese version of the IAT and examine its reliability and validity (face, concurrent, and factorial). Secondly, to give an overview of the extent of the problems caused by IA in the surveyed sample along with related socio-demographic correlates.

## METHODS

### Sample, procedure and participants

The present study took place in Lisbon and included 593 adolescents and young adult students from different regions of the country. Participants were recruited from (i) two high schools and one university in Lisbon (offline paper-and-pencil method), and (ii) an online survey that was available and was disseminated via snowball sampling method. The paper-and-pencil condition comprised 540 participants (91.1%) while the online survey condition had only 53 participants (8.9%). Despite the low proportion of participants that completed the online survey, it was originally included as an attempt to increase the sample size. For the purpose of selection of the participants, data only included those that reported being Portuguese or having a Portuguese and other (not specified) nationality.

### Measures

*Socio-demographics:* These measures included nationality, gender, relationship status, age, academic year, and engaging in hobbies (excluding any online-related activity).

*Internet Addiction Test:* The IAT comprises 20 items, each of which is rated on a six-point Likert scale: ‘does not apply’ (0), ‘rarely’ (1), ‘occasionally’ (2), ‘frequently’ (3), ‘often’ (4), and ‘always’ (5). According to the author (Young, 2011), the test measures the extent of a person’s involvement with the Internet and classifies addictive behaviour in terms of mild, moderate, and severe impairment. To obtain the IAT’s total score, the researcher just needs to sum the scores for each response provided by the participant (Young, 2011). Although several authors have used different cut-off points for diagnosing IA, no clinical or empirical cut-off for the IAT has yet been validated. However, the author did suggest two different cut-off points criteria based on her opinion (Young, 1998b, 2011).

Further procedures were taken in order to achieve IAT’s translation, back translation, and face validity when adapting the instrument to Portuguese. More specifically, for the initial translation of the instrument from English to Portuguese, four independent translators were hired (i.e., two psychologists and two Portuguese/English speakers). Consequently, four Portuguese versions of the IAT were created from the original version. The researchers then analysed each one of the four versions and matched them in a single Portuguese version with all the amendments made to the item wording. After this process, a native English speaker that was proficient in Portuguese was recruited to do a back translation from Portuguese to English for a comparison between the back translated version and the original one.

Validity (i.e., testing what the measure is supposed to measure) can be assessed in several ways (e.g., face, content, concurrent, predictive, factorial) (Shrock & Coscarelli, 2008). To assess the test’s face validity, a careful and analytic discussion between the researchers was carried out. This concluded that the final Portuguese version created from the previous matching was satisfactory for the purpose of the present study as it appeared to reflect the content of the original test and also have good face validity. Additionally, face validity was further assessed directly with five possible test-takers where they were asked to tell the researchers what they thought each item was assessing after reading them.

*Beck Depression Inventory-II:* The Portuguese translation (Ponciano, Cardoso & Pereira, 2005) of the BDI-II (Beck, Steer & Brown, 1996) is a revised 21-item test with four response options per item that range from absence of that symptom (0) to severe or persistent expression of that symptom (3) in the past 2 weeks. Each item represents a particular symptom of depression and corresponds to the diagnostic criteria listed in the DSM-IV (APA, 1994). Total score is categorised as minimal depression (0–13), mild depression (14–19), moderate depression (20–28), or severe depression (29–63). Estimates of internal consistency reliability have ranged from .88 to .94 for clinical and nonclinical adults (Arnau, Meagher, Norris & Bramson, 2001; Beck et al., 1996; Seignourel, Green & Schmitz, 2008). In the present study, reliability was .84 for the whole sample. According to several studies, IA has been systematically associated with depression (e.g., Dalbudak et al., 2012; Ha et al., 2007; Hinić, Mihajlović & Đukić-Dejanović, 2010; Kim et al., 2006; Lam & Peng, 2010; Young & Rodgers, 1998). Therefore, by investigating the relationship of the construct (i.e., IA) to other similar measures of similar constructs (or by correlating scores on the IAT with variables that have been empirically linked with IA) would further allow us to ascertain concurrent validity of the IAT. Thus, in the present study concurrent validity was assessed by comparison of the scores on the IAT with the severity of depressive symptoms. If IA is related to this concept (i.e., depressive symptoms) in the expected direction, this would further validate its practical use as a construct.

### Statistical analysis

Statistical analysis comprised several steps involving a (i) descriptive analysis of the data, (ii) confirmatory factor analysis (CFA), (iii) assessment of concurrent validity and reliability, (iv) within sample prevalence rates of IA, and (v) analyses of the correlates between IA and socio-demographic characteristics of the sample. All the analyses were made using *IBM SPSS Statistics Version 21,* with the exception of the CFA which was performed using MPLUS 6.1 (Muthén & Muthén, 2011) with robust maximum-likelihood estimation (MLR). As for the CFA, in order to address model fit, a *p* value of chi-square smaller than .05 for test of close fit was considered. Moreover, other fit indices included comparative fit index (CFI), Tucker–Lewis Fit Index (TLI), root mean square residual (SRMR). An acceptable fit would be translated by a CFI greater than .90 and a RMSEA value smaller than .08, whereas a good fit is expressed by a CFI value higher than .95 and a RMSEA value close to .06 (Byrne, 2013; Hu & Bentler, 1999).

### Ethics

The study procedures were carried out in accordance with the Declaration of Helsinki. The Institutional Review Board of the Instituto Superior de Psicologia Aplicada (ISPA-IU) and the Portuguese Ministry of Education approved the study. All subjects were informed about the study and all provided written informed consent. Parental written informed consent was provided by parents for those younger than 18 years old.

## RESULTS

### Descriptive statistics

Of the total participants (N = 593, 94% Portuguese and 5.7% Portuguese and Other Nationality), 69% of participants (n = 409) were female. Ages varied greatly (Mean_age_ = 19 years,

SD = 3.61) from 15 years (minimum) to 39 (maximum) years. The majority of the participants were not in a relationship (n = 349, 58.9%). Moreover, 56% (n = 332) did not engage in any hobbies. In terms of participants’ academic year, 14.3% (n = 85) were in the first year, 9.1% (n = 54) were in the second year, and 19.7% (n = 117) in third year of secondary school respectively. Regarding the university students, 18.4% were first year students (n =109), 15.7% second year students (n = 93), 16.2% third year students (n = 96), 2.7% fourth year students (n =16), and 3.9% fifth year students (n = 23).

### Confirmatory factor analysis

Following the findings of previous similar studies using the IAT (Hawi, 2013; Khazaal et al., 2008; Panayides & Walker, 2012), a uni-factorial model for the IAT was tested. A confirmatory factor analysis (CFA) was performed to the test the viability of the uni-factorial structure of the IAT. The latent construct was IA – which was not directly observed – as it was considered the endogenous variable. On the other hand, the 20 items were considered the exogenous variables used to measure participant’s IA level. The analysis of the first-order model provided an acceptable model fit for the IAT. More specifically, c^2^ (162, N = 593) = 373.6, *p* < .0001; CFI = .926; TLI = .913; RMSEA = .047 (90%CI: .041–.053), *p*close = .785; SRMR .050. In this model, all factor loadings were acceptable with the exception of the loading on Item 3 and Item 7 (see Table 1). In order to address model fit, the authors co-varied certain residuals (i.e., e8 and e6; e1 and e2) within the factor due to systematic covariance. In sum, the results obtained from the CFA were acceptable and indicate an overall acceptable model.

**Table 1.** Obtained loadings from the CFA and reliability for the 20 IAT items

	*How often…*	Loadings
1.	Do you find that you stay on-line longer than you intended?	.50
2.	Do you neglect household chores to spend more time on-line?	.56
**3.**	**Do you prefer the excitement of the Internet to intimacy with your partner?**	**.19**
4.	Do you form new relationships with fellow on-line users?	.48
5.	Do others in your life complain to you about the amount of time you spend on-line?	.63
6.	Do your grades or school work suffer because of the amount of time you spend on-line?	.59
**7.**	**Do you check your e-mail before something else that you need to do?**	**.27**
8.	Does your job performance or productivity suffer because of the Internet?	.56
9.	Do you become defensive or secretive when anyone asks you what you do on-line?	.48
10.	Do you block out disturbing thoughts about your life with soothing thoughts of the Internet?	.57
11.	Do you find yourself anticipating when you will go on-line again?	.66
12.	Do you fear that life without the Internet would be boring, empty, and joyless?	.53
13.	Do you snap, yell, or act annoyed if someone bothers you while you are on-line?	.63
14.	Do you lose sleep due to late-night log-ins?	.61
15.	Do you feel preoccupied with the Internet when off-line, or fantasize about being on-line?	.69
16.	Do you find yourself saying “just a few more minutes” when on-line?	.65
17.	Do you try to cut down the amount of time you spend on-line and fail?	.66
18.	Do you try to hide how long you’ve been on-line?	.58
19.	Do you choose to spend more time on-line over going out with others?	.51
20.	Do you feel depressed, moody, or nervous when you are off-line, which goes away once you are back on-line?	.62
Scale Reliability (α)		.90

### Concurrent validity and reliability

Concurrent validity was assessed by comparing the BDI-II scores with the IAT scores. Moreover, concurrent validity was achieved by a statistically significant and positive correlation between the two variables (*r* = .82, *p* < .0001). The internal consistency of the IAT was measured using Cronbach’s alpha, and is the most popular coefficient of reliability measure (Tavakol & Dennick, 2011). Theoretically, it varies from 0 to 1 and should be above .7 (DeVellis, 2003). In this study, the data collected for the Portuguese version of the IAT produced highly consistent internal reliability (α = .90) and considered excellent because α is ≥ .90 (George & Mallery, 2003).

### Within sample prevalence rates of Internet addiction

When the IAT was developed, Young (1998b) suggested a specific cut-off point that distinguished between three types of Internet user according to different level of impairments caused by Internet usage. However, many years later, new cut-off points were proposed by the author (i.e., Young, 2011), but this time four types of Internet user were distinguished in terms of the impairments caused by the level of Internet usage. It is worth noting that both proposed cut-offs are speculative because no actual empirical or clinical studies were conducted in order to ascertain the actual cut-off points of this test. Consequently, these results must be cautiously interpreted in terms of its external validity as it may only concern to the sample used in the present study and not the general population.

Adopting the initially proposed cut-off criteria (Young, 1998b) – 20–39 = average user; 40–69 = a person that has frequent problems because of his/her Internet usage; 70–100 = Internet addicts – 53% of the sample would be classed as average online users (n = 314), while another 33.9% might be experiencing some degree of problems caused by their Internet usage (n = 201). Only 1.2% could be classed as Internet addicts using this criteria (n = 7). Conversely, when using the second proposed cut-off (Young, 2011) – 0–30 = normal range; 31–49 = mildly addicted; 50–79 = moderately addicted; and 80–100 = severely addicted – 35.1% are normal Internet users (n = 208), 44% are mildly addicted (n = 261), and 16% are moderately addicted (n = 95). Contrary to the results found using the first proposed cut-off criteria, the second cut-off score revealed none of the participants could be classed as an Internet addict. However, the number of participants at risk of IA appears to be very high when considering those belonging to the mildly and moderately addicted groups (i.e., 60%, n = 356).

### Socio-demographic correlates and Internet addiction

In regard to gender, males and females differed significantly in their IAT scores, (U = 25.282, *p* < .001). Age and IAT scores were also significantly correlated (*r_s_* = –.257, *p* < .0001). Furthermore, age explained 6.6% of the variance of IA. Relationship status was also examined. Results indicated that the groups (i.e., being in a relationship vs. not being in a relationship) differed significantly in relation to their IAT scores (*t*[561] = 2.049, *p* = .041). Participants that were in a relationship scored lower on IAT than those who were not (*r* = –.086, *p* = .0041). Moreover, this variable accounted for .74% of the total variance of IA. In relation to engaging in hobbies, this variable did not seem to have any statistically significant effect of IAT scores (*t*[562] = .407, *p* = .684) in the present sample.

Finally, there was a significant effect for participant’s educational stage (*F*[7, 556] = 9.06, *p* < .0001) on IAT scores, with secondary school students having higher scores than university students. Post-hoc comparisons using Tukey HSD test further suggested that IAT mean scores for first year secondary students (Mean_IAT_ = 12.94, SD = 9.18) were significantly different than secondary school students in their third year (Mean_IAT_ = 9.96, SD = 7.67, *p* = .003), in addition to university students in their first year (Mean_IAT_ = 10.14, SD = 6.17, *p* < .0001), second year (Mean_IAT_ = 8.83, SD = 6.13, *p* < .0001), third year (Mean_IAT_ = 8.79, SD = 6.24, *p* < .0001) and fifth year (Mean_IAT_ = 8.48, SD = 8.66, *p* < .0001). Additionally, IAT mean scores also significantly differed between third year secondary school students (Mean_IAT_ = 8.79, SD = 6.24) and first year university students (Mean_IAT_ = 10.14, SD = 6.17, *p* = .040). These findings contrast those from other studies (Demetrovics, Szeredi & Rózsa, 2008) using the PIUQ for assessing IA. In sum, secondary school students displayed higher levels of IA (Mean_IAT_ = 40.55, SD = 14.57) in comparison with university students (Mean_IAT_ = 33.18, SD = 11.91) as observed by mean comparison tests (*t*[434.16] = 6.368, *p* < .0001).

## DISCUSSION

This study attempted to give a preliminary overview in regard to the phenomenon of Internet Addiction (IA) in Portugal. More specifically, IA was assessed in a sample of students from secondary schools and universities across the country. Considering the objectives of the present study, the authors explored the extent of IA and its socio-demographic correlates in the sample while also exploring the psycho-metric properties of a Portuguese version of the IAT. The study found only 1.2% of participants (n = 7) were classed as Internet addicts using Young’s original (1998b) criteria. This is in line with other studies using the IAT that have reported similar prevalence rates ranging from .6% in one study (Lam, Peng, Mai & Jing, 2009) to .9% (Yoo et al., 2004).

In regard to gender, males and females differed significantly in their IAT scores, In line with previous studies (e.g., Esen & Gündogdu, 2010; Liberatore et al., 2011; Ko et al., 2006; Yoo et al., 2004), males scored significantly higher on IAT in comparison to female respondents (*r_pb_* = –.185, *p* < .001). However, other studies did not find significant differences regarding gender and IA levels using the PIUQ (Demetrovics et al., 2008). Moreover, this variable appeared to account for 3.4% of the total variance of IA in the present sample. Despite the gender differences observed in relation to IA in the present sample, this variable only accounted for a small percentage (i.e., 3.4%) of the IA levels. Similar to other studies (Demetrovics et al., 2008; Morrison & Gore, 2010; Ni, Yan, Chen & Liu, 2009; Smahel, Brown & Blinka, 2012), age was also an important factor for IA with younger people displaying higher levels of IA. Results also showed that age and IAT scores were also significantly correlated. Similarly with previous studies (Demetrovics et al., 2008; Morrison & Gore, 2010; Ni et al., 2009; Smahel et al., 2012), younger people scored higher on IAT than older people.

To the authors’ knowledge, no study has addressed the potential protective factors of romantic relationships regarding IA. In the present study, people that reported being engaged in a romantic relationship displayed lower levels of IA. Moreover, having an offline hobby did not constitute as a protective factor for IA as initially thought by the authors. This should be further investigated in future studies as hobbies may play an important role in helping prevent some substance use disorders (Hu, Sekine, Gaina, Nasermoadelli & Sadanobu, 2007), and contributing to a general healthy lifestyle (Inoue et al., 2010).

Regarding participants’ academic year, the present findings are in line with those from other studies (e.g., Demetrovics et al., 2008) as secondary school students displayed higher levels of IA compared to university students. Because secondary school students are generally younger than university students, this association might be partly explained by the age.

Previous investigations into IAT’s internal consistency have found good results across several studies, ranging from .54–.82 (Widyanto & McMurran, 2004), .92 (Hawi, 2013), .93 (Khazaal et al., 2008), .83–.91 (Jelenchick et al., 2012), .90 (Milani, Osualdella & Di Blasio, 2009) and good bi-weekly test–retest reliability (i.e., r = .85) in another study (Yang, Choe, Baity, Lee & Cho, 2005). Similar to these results, the adapted version of the IAT in the present study was also found to have a good internal consistency (a = .90).

In terms of concurrent validity, the results obtained were also satisfactory and empirically warranted. Moreover, the CFA results supported the authors’ model as evidenced by an acceptable model fit. Despite the fact that Item 3 (i.e., *How often do you prefer the excitement of the Internet to intimacy with your partner?*) and Item 7 (i.e., *How often do you check your e-mail before something else that you need to do?*) resulted in problematic factor loadings (as similar to other findings, e.g., Law & Chang, 2007), this does not compromise future usage of the test as long as researchers take into consideration two aspects.

Firstly, Item 3’s low loading might be partially explained by the sample characteristics as more than half of them (60%) were not in a relationship, therefore this item would not apply to their situation. Secondly, Item 7’s low factor loading might be attributed to the major changes that technology in general and the Internet itself underwent. Nowadays, (as opposed to 1998 when IAT was developed), a large proportion of people can check their e-mails almost anywhere via their mobile devices, which makes this situation not as representative of IA as it used to be in the past. Future research should address this by either proper item rewording or removing the item as long as the model is supported. In conclusion, the psychometric study of the IAT in the present sample provided satisfactory empirical evidence for its future use despite the low loadings on Items 3 and 7.

Although the results showed an acceptable model fit, the authors had to covary the residuals within factor from e8 (item 8) and e6 (item 6), and e1 (item 1) and e2 (item 2) due to systematic covariance, possibly stemming from (i) the sequence in which the questions were answered by the participants (e.g., items 1 and 2) and/or due to the (ii) similarities in terms of item wording (e.g., items 8 and 6). However, without adding the present covariances between the errors of the items, the model fit would be lower than desired.

The factor structure of the IAT has been subject of an extensive debate. Nevertheless, a uni-dimensional solution seemed to be suitable in this study and is thus in line with the findings of similar previous studies (Hawi, 2013; Khazaal et al., 2008; Panayides & Walker, 2012). However, it is worth noting that many studies have reported different results with IAT ranging from one (Hawi, 2013; Khazaal et al., 2008; Panayides & Walker, 2012), two (Jelenchick et al., 2012; Korkeila, Kaarlas, Jaaskelainen, Vahlberg & Taiminen, 2010; Pawlikowski, Altstotter-Gleich & Brand, 2013), three (Law & Chang, 2008; Widyanto, Griffiths & Brunsden, 2011), and (up to) even six factors (Ferrarro et al., 2007; Widyanto & McMurran, 2004). This does not appear to help researchers in the field, as it reflects the lack of conceptual inconsistency and controversy in the literature regarding the IAT and even IA itself when assessed with this particular instrument.

A possible explanation for these rather inconclusive outcomes may lie in the fact that – from a methodological point of view – most of these studies come from different countries, where cultural effects may be playing an important role in terms of social representations of the Internet itself and its misuse. Furthermore, many of these studies have widely different sample sizes, ranging from less than 100 (Widyanto & McMurran, 2004) to almost 2000 individuals (Watters, Keefer, Kloosterman, Summerfeldt & Parker, 2013) that may account for some of the outlined discrepancies in regard to the IAT’s factorial structure.

The reported findings also suggested a low prevalence of IA (1.2%) in the present sample. These results should be cautiously interpreted and not generalised to the general population because of several issues. Firstly, the sample is not representative of the entire population, thus the present results may only concern the students included in the study. Secondly, as previously shown, there appear to be important inconsistencies with the cut-off researchers should use, since the original author did not propose any empirical or clinical based cut-off. Thirdly, several researchers have used different cut-off points to ascertain levels of IA. This makes future comparisons not as straightforward as it should be when using the same instrument.

Despite these potential limitations, our findings support the notion that IA is a condition worthy of future studies because of the well documented harms that it may pose to people. However, IA in the present study was not as widespread in the participants sampled as demonstrated by the low prevalence rates using the initial proposed cut-off points (Young, 1998b). However, when using the later proposed cut-off points (Young, 2011) the proportion of participants at risk of addiction was relatively high (60%) despite no participants exhibiting addiction levels as operationalized by scoring at the highest level of between 80 to 100 points on the IAT.

The low prevalence rate of IA reported here suggests two possible issues in the present study: (i) generalised IA as conceptualised by IAT does not appear to be a major endemic problem in the present sample (although this does not indicate that IA is not a real source of problems and negative outcomes to people that suffer from their Internet misuse); and/or (ii) the low prevalence rates might indirectly imply that some specific properties of Internet use itself were overlooked (i.e., specific applications and/or online behaviours) and omitted individuals that suffered from other online addictions (e.g., to online gambling or online gaming, etc.).

In terms of limitations, the IAT itself has a number of methodological shortcomings. These may account for some of the present limitations of this study. Firstly, as suggested by some early pioneers in the IA field (e.g., Griffiths, 1999), there are important issues concerning Young’s proposed criteria of IA that should be taken into account, such as the fact that they have no temporal dimension and take no account of the context of Internet use (Griffiths, 1999). Moreover, the evolving IA conceptualisations made over time by many researchers, the IAT might be accused of being outdated in some aspects as Internet use itself greatly evolved since 1998. For instance, Item 7 (i.e., *How often do you check your e-mail before something else that you need to do?*) is a good example as it illustrates an old-fashioned way of checking e-mails that may not be practiced by the majority of people as it used to as they can now receive push messages from their portable devices (i.e., smartphones, tablets) implying no direct access to their e-mail website at all.

Secondly, item wording in the IAT does not appear to reflect a rigorous psychometric process of item creation. A thorough analysis of the item-writing guidelines is far beyond the scope of this paper. However, as a general rule of thumb, every item should reflect specific content and a single specific mental behaviour (Haladyna, Downing & Rodriguez, 2002). This does not appear to be the case in a majority of the IAT’s items (e.g., *How often do you feel depressed, moody, or nervous when you are offline, which goes away once you are back online?*). While for some people IA withdrawal effects can be described in terms of unique depressive symptoms, for others it does not necessarily have to be that way, as they could feel moody but not nervous. Thirdly, because the present study utilised a convenience sample the majority of which were adolescent or young adult women, generalisations to the general population should not be made.

As a concluding note for future studies, IA should not only be measured with instruments that are psychometrically sound (i.e., valid and reliable), but also seek to use a more unified measurement tool based on the latest criteria for IGD as in the DSM-5 (Griffiths et al., 2014). This should largely contribute to the development and recognition of IA as an independent clinical entity. Finally, the present study is important for a number of different reasons. Firstly, it serves as a basis for introducing the topic of IA to Portuguese clinicians that are interested in this area and want to assess it. Secondly, academic researchers may use the newly validated instrument to further develop the knowledge of IA in Portugal and refine the assessment of it by criticising and improving on this initial validation. Thirdly, from a societal viewpoint, this study can contribute positively to a better understanding of the basic aspects of IA in the Portuguese community since many health practitioners are starting to realise that – for some individuals – Internet use may pose a risk.

## Figures and Tables

**Table:** Young’s Internet Addiction Test – Teste de Dependência ä Internet^1,2,3^

*Items*	*Question:* Com que frequência…
Item 1	Ficas online mais tempo do que pretendias?
Item 2	Deixas de fazer as tarefas em casa para poderes ficar mais tempo online?
Item 3	Preferes a excitação da Internet ä intimidade com o teu(tua) namorado(a)?
Item 4	Crias novas relações com outros utilizadores online?
Item 5	As outras pessoas se queixam em relação ä quantidade de tempo que passas online?
Item 6	As tuas notas ou trabalhos escolares são prejudicados devido ä quantidade de tempo que passas online?
Item 7	Verificas o teu e-mail (ou sites como o facebook ou twitter) antes de fazeres qualquer outra coisa que precisas?
Item 8	O teu desempenho ou produtividade no trabalho são prejudicados por causa da Internet?
Item 9	Te tornas defensivo(a) ou guardas segredo quando alguém te pergunta o que estás a fazer online?
Item 10	Bloqueias pensamentos perturbadores sobre a tua vida com pensamentos calmantes da Internet?
Item 11	Dás por ti a pensar sobre quando irás estar online novamente?
Item 12	Receias que a vida sem Internet seria chata, vazia e sem graça?
Item 13	É que explodes, gritas ou ficas irritado(a) quando alguém te incomoda quando estás online?
Item 14	Perdes o sono por estares online até tarde durante a noite?
Item 15	Te sentes preocupado(a) com a Internet quando estás desconectado(a) ou fantasias estar online?
Item 16	Dás por ti a dizer “só mais alguns minutos” quando estás online?
Item 17	Tentas reduzir a quantidade de tempo que passas online e não consegues?
Item 18	Tentas esconder a quantidade de tempo que passaste online?
Item 19	Preferes ficar mais tempo online do que ir sair com outras pessoas?
Item 20	É que te sentes deprimido(a), mal-humorado(a) ou nervoso(a) quando estás desconectado(a) e, deixas de estar assim quando entras online novamente?

^1^ Instruç^es: As perguntas que se seguirão, irão avaliar alguns dos teus hábitos em relação ao uso do computador, mais concretamente da Internet. Por isso, ao responder äs perguntas deves ter em consideração **SOMENTE** o tempo que passas online por **MOTIVOS RECREACIONAIS** ou **DIVERSÃO**, e não o tempo gasto na Internet com trabalhos escolares ou laborais.

^2^ **Escala de 6-pontos:** 0: Não Aplicável; 1: Nunca; 2: Raramente; 3: Ocasionalmente; 4: Várias Vezes; 5: Sempre.

^3^ O termo dependência é preferível ao “adição” uma vez que este último remete para operaç^es matemáticas de soma e não ao comportamento em si.
